# Comparatively low attendance during Human Papillomavirus catch-up vaccination among teenage girls in the Netherlands: Insights from a behavioral survey among parents

**DOI:** 10.1186/1471-2458-12-498

**Published:** 2012-07-02

**Authors:** Giedre Gefenaite, Marieke Smit, Hans W Nijman, Adriana Tami, Ingrid H Drijfhout, Astrid Pascal, Maarten J Postma, Bert A Wolters, Johannes J M van Delden, Jan C Wilschut, Eelko Hak

**Affiliations:** 1 Department of Pharmacy, Pharmacoepidemiology & Pharmacoeconomics (PE2), University of Groningen, Groningen, the Netherlands; 2 Department of Epidemiology, University of Groningen, University Medical Center Groningen, Groningen, the Netherlands; 3 Department of Obstetrics & Gynaecology, University Medical Center Groningen, Groningen, the Netherlands; 4 Department of Medical Microbiology, Molecular Virology Section, University Medical Center Groningen, Groningen, the Netherlands; 5 Institute for Public Health and the Environment (RIVM), Bilthoven, the Netherlands; 6 Municipal Health Service Groningen, Groningen, the Netherlands; 7 University Medical Center Utrecht, Julius Center, Utrecht, the Netherlands

## Abstract

**Background:**

The Dutch Human Papillomavirus (HPV) catch-up vaccination program in 2009 appeared less successful than expected. We aimed to identify the most important determinants of refusing the vaccination.

**Methods:**

Two thousand parents of girls born in 1996 targeted for HPV vaccination received an invitation letter to participate in a questionnaire study. Two study groups were defined: the first group consisted of parents of girls who had accepted the vaccine and already received the first dose of HPV vaccination. The second group consisted of parents whose daughters were not vaccinated. The questionnaire consisted of a broad spectrum of possible determinants that were revealed after literature search and discussions with the stakeholders.

**Results:**

Four hundred sixty nine questionnaires (24%) were returned, 307 (31%) from those who accepted and 162 (16%) from those who declined the vaccine. The decision not to accept the vaccine was largely determined by: (i) perception that the information provided by the government about the vaccine was limited or biased (OR 13.27); (ii) limited trust, that the government would stop the vaccination program if there were serious side effects (OR 9.95); (iii) lack of knowledge about the effectiveness of the vaccine (OR 7.67); (iv) concerns about the side effects of the vaccine (OR 4.94); (v) lack of conviction that HPV can be extremely harmful (OR 3.78); (vi) perception that the government is strongly influenced by vaccine producers (OR 3.54); and (vii) religious convictions (OR 2.18).

**Conclusions:**

This study revealed several determinants for HPV vaccination uptake after implementation of the HPV vaccine for adolescent girls. These determinants should be taken into consideration in order to successfully implement HPV vaccination into National Immunization Programs.

## Background

Based on the recommendations by the Dutch Health Council (DHC) in March 2008 [1], the Dutch government approved implementation of the Human Papillomavirus (HPV) vaccination as part of the National Immunization Program (NIP). The vaccine to be used was Cervarix™ (GlaxoSmithKline), which is a bivalent vaccine against HPV16 and HPV18, and consists of three doses administered at baseline, one and six months [2]. The vaccine was mainly targeted at 12-year-old girls (1997 birth cohort) and a catch-up vaccination program was planned for 13- to16-year-old girls (1993–1996 birth cohort) [1]. The catch-up vaccination campaign started in March 2009, and the regular vaccination campaign began in 2010. For all targeted girls the vaccination campaign was free of charge. The girls received a personal invitation letter with an information leaflet and were invited to visit local vaccination sessions [3]. Although no permission of the parents was required, the girls were advised to discuss the information and their decision regarding HPV vaccination with parents or other family members [4].

Ultimately, slightly above fifty percent (52.3%) of the targeted catch-up cohort received the HPV vaccine [5]. The vaccination rates varied by age, from 49.0% in 1993 birth cohort to 54.2% in, 1996 birth cohort [5]. By February 2011, similar vaccination rates (52.5%) were observed in the 1997 birth cohort [5]. One suggested reason for such a low HPV vaccination uptake, as compared to the normal attendance rates for the regular vaccinations of the NIP of 90% [5], was critical reporting in the media, which accused the government of emphasizing the advantages rather than the potential disadvantages of the vaccine. Other reasons, such as the relative novelty of the vaccine, the fact that it was the first vaccine for a sexually transmitted infection and the first vaccine for girls only, and its unknown effectiveness in preventing cancer could have engendered distrust in the parents as well. Another possible factor in declining HPV vaccination or vaccinations in general, could be religious beliefs. In the Netherlands, the overall vaccination coverage is much lower in the so-called Bible-belt region where relatively many people decline vaccinations because of their religious convictions (http://www.rivm.nl/en/infectious-diseases/topics/nip/).

Several Dutch studies have already assessed the knowledge about and the willingness to receive the HPV vaccine. These studies have shown that 56%–88% of the respondents would be willing to receive or have their daughter receive the HPV vaccine [6,7]. Several studies from other countries that assessed parental attitudes towards HPV vaccination showed similar rates of intention to vaccinate (70–80%) [8-10]. Since actual behavior may be different from intentions, we designed a study which aimed to assess a broad range of demographic, behavioral, and organizational determinants, and knowledge and concerns that were influential in parents’ decision to either accept or decline the vaccination for their daughters during the HPV vaccination campaign. Although in the catch-up vaccination campaign 13- to 16-years-old girls were invited to receive HPV vaccinations, only the girls of 13 years of age were invited to our study as with regard to age their parents resembled the parents of the 12-years-old girls the best.

## Methods

In July 2009, four months after the HPV vaccination catch-up program for 13- to 16-year-old girls was initiated, we randomly selected parents of girls born in 1996 who had received a call for the HPV vaccine in the Northern provinces of the Netherlands. Two study groups were defined. The first group consisted of parents of girls who had accepted the vaccine and already received the first dose of HPV vaccination. The second group consisted of vaccine decliners. The two groups, including 1000 parents each, were randomly sampled from the vaccination register. On behalf of the researchers, the Institute for Public Health and the Environment (RIVM) sent 2000 invitations to eligible parents to ask if they would be willing to participate in the study. Parents who agreed to enroll in the study returned the response cards with a positive reply and parents who were willing to participate received a paper questionnaire in Dutch (the English version added in Additional file 1). After three weeks a reminder to fill out the questionnaire was sent.

The study was conducted in accordance with the Dutch Law for the Protection of Personal Data (Wet Bescherming Persoonsgegevens) and the Declaration of Helsinki (http://www.wma.net/e/policy/b3.htm). No medical ethical committee approval was required.

We used an anonymous, self-administered questionnaire to identify the determinants for not accepting the HPV vaccination. In a stakeholders meeting with professionals involved in the HPV campaign, (a gynecologic oncologist, a sexologist, a doctor of the Municipal Health Service, a regional manager of coordination programs at the center for infectious disease control, an epidemiologist, and municipal health advisors), possible determinants for the uptake of the HPV vaccination were explored. In addition, a review of the literature, a stakeholder analysis, and questionnaires previously developed by our and other research groups [9] were used to construct a new questionnaire. The parents were asked to fill out the questionnaire (in Dutch) on behalf of their daughter who had received a call for the HPV vaccination. Parents could also express their attitudes towards HPV vaccination in a free text.

The demographical determinants of the parents included gender, age, marital status, educational level (none, primary, prevocational, secondary, pre-university, higher-professional, university), religion (Catholic, Protestant, Islam or other) and country of birth. We also assessed the participation in a cervical screening program. We asked whether a participant knew someone with an abnormal cervical smear or cervical cancer in his/her family or circle of acquaintances. Items reflecting behavioral determinants were based on health behavior criteria according to the “Health Belief Model” and “Behavioral Intention Model” [11,12]. We formulated questions based on five of the six domains in the Health Belief Model; perceived susceptibility, perceived severity, perceived benefits, perceived barriers and cues to action. We posed questions on two more domains that exist in the Behavioral Intention Model; attitudes and social influences. We also posed questions about parents’ knowledge concerning HPV and cervical cancer, information services and sources that influenced decision-making, trust in the government, vaccination concerns, age-related items, financial issues, intention to accept the vaccination later, involvement of their daughter in the final decision and future acceptance of other vaccines.

We dichotomized the following variables: education (high (university, higher professional)/other), being religious (yes/no), country of birth (the Netherlands/other), knowing someone with an abnormal cervical smear or cervical cancer in his/her family or circle of acquaintances (yes/no), and participation in cervical screening program (yes/no). The variables assessed with the four- and five-point Likert scale were dichotomized according to the degree of agreement with the proposition, (4–5 [agree - strongly agree]) for the indicator group, and (1–3 [strongly disagree, disagree and disagree nor agree]) for the reference group.

Knowledge about HPV and HPV vaccination was assessed by using 10 statements (true/false/does not know), see Appendix 1. The mean knowledge score was calculated.

The primary outcome was the uptake of the first dose of the HPV vaccine obtained from RIVM.

All determinants with a p-value lower or equal to 0.10 in the univariate analyses were used in the multivariate analysis. We used all determinants with a p-value of 0.05 or lower to construct a final logistic regression model. We calculated odds ratios (OR) and 95% confidence intervals (95% CI) as measures of associations. The area under the curve (AUC) for the receiver operating characteristic (ROC) with its 95% CI was calculated. The statistical analysis was performed using SPSS for Windows (version 16.0; SPSS, Inc Chicago Illinois).

## Results

Of the 2000 parents approached by the administrative offices of the RIVM, 863 parents responded and 609 parents were willing to co-operate. Four hundred sixty nine parents returned the questionnaire. Overall the response rate was 24% (469/2000), 31% (307) in the group who received the vaccine versus 16% (162) in the group who did not. Of the respondents 93% (435/468) were female, and mean age was 44 years (range 35–55 years). The mean score for knowledge about HPV and cervical cancer was 5.65/10 correct answers, 5.54 and 5.88 (*p = .09*) in those accepting and refusing the vaccine respectively.

The HPV vaccine was accepted by 66% of the respondents’ daughters while 34% (162/469) declined it. The majority (96%) of the girls received the second dose.

When the parents could express their attitudes towards the HPV vaccination, no new determinants of refusing the vaccine were encountered.

## Determinants associated with not accepting the HPV vaccination

The results from the univariate analysis to determine the association between the demographic determinants and not accepting the HPV vaccine are shown in Table 1. Being religious was a strong demographic determinant for declining HPV vaccination.

**Table 1:** Univariate analysis: demographical determinants and declining the HPV vaccination

**Determinants**	**Vaccinated n = 307**	**Not vaccinated n = 162**	**Odds ratio (95% CI)**	**p-value**
Educational level, high	74/305 (24.3%)	51/162 (31.5%)	1.43 (0.94–2.19)	0.09
Religious, yes	152/306 (49.7%)	113/162 (69.8%)	2.34 (1.56–3.50)	<0.001
Country of birth, the Netherlands	298/306 (97.4%)	158/162 (97.5%)	1.06 (0.31–3.58)	0.93
Knowing someone with an abnormal cervical smear or cervical cancer in his/her family or acquaintances, no	181/306 (59.2%)	83/162 (51.2%)	0.73 (0.50–1.06)	0.10
Participation in cervical screening, no	23/306 (7.5%)	10/162 (6.2%)	0.81 (0.37–1.75)	0.59
Regular NIP vaccinations, no	0/304 (0%)	21/162 (13%)	.32 (.28–.36)	<0.001

Several behavioral determinants were associated with declining the HPV vaccination (Table 2). These included the conviction of the parents that their daughter would not get infected with HPV, lack of belief that HPV can be extremely harmful, judgment that it would be unlikely that their daughter might get cervical cancer in the future, perception that vaccinations are not effective in preventing disease, and conviction that HPV is not sufficiently serious to warrant vaccination.

**Table 2:** Univariate analysis: behavioral determinants and declining the HPV vaccination

**Determinants**	**Vaccinated n = 307**	**Not vaccinated n = 162**	**Odds ratio (95% CI)**	**p-value**
It’s not likely that my daughter gets infected with HPV some day	81/306 (26.5%)	59/162 (36.4%)	1.59 (1.06–2.39)	0.03
I don’t believe HPV can be extremely harmful	37/304 (12.2%)	39/162 (24.1%)	2.29 (1.39–3.77)	0.001
It’s not possible that my daughter gets infected with HPV some day	27/305 (8.9%)	13/160 (8.1%)	0.91 (0.46–1.82)	0.79
I don’t believe HPV can cause cervical cancer	24/305 (7.9%)	7/161 (4.3%)	0.53 (0.22–1.26)	0.15
It’s not possible that my daughter gets cervical cancer in the future	15/306 (4.9%)	3/162 (1.9%)	0.37 (0.10–1.28)	0.10
I don’t believe that cervical cancer is a serious disease	4/305 (1.3%)	1/162 (0.6%)	0.47 (0.05–4.22)	0.49
It’s not likely that my daughter gets cervical cancer in the future	80/306 (26.1%)	62/160 (38.8%)	1.79 (1.19–2.69)	0.01
Cervical cancer is not something I’m worried about right now for my daughter	117/305 (38.4%)	60/162 (37%)	0.95 (0.64–1.40)	0.78
Vaccines aren’t effective in preventing diseases	12/303 (4.0%)	25/153 (16.3%)	4.74 (2.31–9.72)	<0.001
HPV is not that serious to get vaccinated for	6/302 (2%)	24/155 (15.5%)	9.04 (3.61–22.63)	<0.001

Many determinants regarding the knowledge, and concerns about the safety of the vaccine and organizational issues related to government and information services were associated with declining HPV vaccination (Table 3).

**Table 3:** Univariate analysis: knowledge, concerns, organisational determinants and declining the HPV vaccination

**Determinants**	**Vaccinated n = 307 (65.5%)**	**Not vaccinated n = 162 (34.5%)**	**Odds ratio (95% CI)**	**p-value**
I’m very worried about the side effects of the HPV vaccination	79/301 (26.2%)	140/160 (87.5%)	19.67 (11.53–33.56)	<0.001
We know way too little about the effects of the vaccine	172/298 (57.7%)	158/161 (98.1%)	38.58 (12.03–123.71)	<0.001
We don’t know a lot about the side effects of the vaccine	179/298 (60.1%)	155/161 (96.3%)	17.17 (7.36–40.09)	<0.001
It’s not very important that my children receive all their vaccinations	10/305 (3.3%)	51/156 (32.7%)	14.33 (7.02–29.25)	<0.001
I won’t do everything to prevent my daughter getting cervical cancer	7/306 (2.3%)	9/159 (5.7%)	2.56 (0.94–7.02)	0.06
There are already too many vaccines in the Dutch vaccination program	14/306 (4.6%)	26/160 (16.3%)	4.05 (2.05–8.00)	<0.001
I would had have more information to make a good decision	143/305 (46.9%)	136/160 (85.0%)	6.42 (3.94–10.47)	<0.001
I feel I didn’t get enough information to make a good decision	93/303 (30.7%)	95/161 (59%)	3.25 (2.18–4.84)	<0.001
I think the information about the vaccine provided by the government was very limited/biased	115/301 (38.2%)	150/154 (97.4%)	60.65 (21.88–168.17)	<0.001
I think the information about the vaccine provided by the government was not very clear	119/304 (39.1%)	138/160 (86.3%)	9.75 (5.88–16.17)	<0.001
I don’t believe/trust that the government would stop vaccinations if there was evidence of serious side effects	14/305 (4.6%)	87/156 (55.8%)	26.21 (14.06–48.84)	<0.001
The government is strongly influenced by the vaccine producers	49/284 (17.3%)	126/153 (82.4%)	22.38 (13.34–37.54)	<0.001
There weren’t enough locations to get the vaccination	9/306 (2.9%)	6/158 (3.8%)	1.30 (0.46–3.73)	0.62
It wasn’t very clear when my daughter could get the HPV vaccine	12/306 (3.9%)	6/158 (3.8%)	0.97 (0.36–2.63)	0.95
Doctors do not take parents seriously regarding the side effects of vaccinations	35/295 (11.9%)	64/155 (41.3%)	5.22 (3.25–8.41)	<0.001
I think it’s good that the HPV vaccine exists, but not at this age	31/303 (10.2%)	79/152 (52.0%)	9.50 (5.82–15.49)	<0.001
I don’t think my daughter is very capable to make her own decision about accepting the vaccination	208/302 (68.9%)	112/158 (70.9%)	1.10 (0.72–1.68)	0.66
I would get my daughter vaccinated if the vaccine wasn’t only for girls but also for boys	141/303 (46.5%)	12/155 (7.7%)	0.10 (0.05–0.18)	<0.001
Girls who had the HPV vaccination would be more likely to have unprotected sex	5/306 (1.6%)	19/155 (12.3%)	8.38 (3.07–22.92)	<0.001
Having the HPV vaccination might make girls more likely to have sex	21/305 (6.9%)	34/161 (21.1%)	3.62 (2.02–6.49)	<0.001
I would strongly disapprove if my daughter would be sexually active at this age	290/305 (95.1%)	151/162 (93.2%)	0.71 (0.32–1.58)	0.40
Other girls might be vaccinated, but my daughter won’t	7/306 (2.3%)	12/157 (7.6%)	3.54 (1.36–9.17)	0.01

The results from the multivariate analyses indicate that the strongest determinants of not accepting HPV vaccination were: limited information about the vaccine provided by the government, limited trust that the government would stop vaccinations if there were serious side effects and concerns related to vaccine safety, effectiveness and religion (Table 4). The AUC for the final model, including all 7 determinants, was 0.96 (95% CI 0.94–0.97).

**Table 4:** Multivariate analysis: determinants of declining the HPV vaccine.

**Determinants**	**Vaccinated n = 307 (65.5%)**	**Not vaccinated n = 162 (34.5%)**	**OR (95% CI)**	**p-value**
I think the information about the vaccine provided by the government was very limited	103/273 (37.3%)	141/145 (97.2%)	13.74 (3.82–49.46)	<0.001
I have no trust that the government would stop the vaccinations if there was evidence of serious side effects	13/273 (4.8%)	81/145 (55.9%)	10.13 (4.06–25.60)	<0.001
We know way too little about the effects of the vaccine	153/273 (56%)	143/145 (98.6%)	8.34 (1.41–49.50)	0.02
I’m very worried about the side effects of the HPV vaccination	69/273 (25.3%)	127/145 (87.6%)	4.71 (2.13–10.44)	<0.001
I don’t believe HPV can be extremely harmful	34/273 (12.5%)	38/145 (26.2%)	4.19 (1.63–10.82)	0.03
The government is strongly influenced by the vaccine producers	47/273 (17.2%)	120/145 (82.8%)	3.60 (1.74–7.47)	0.001
Religious conviction	133/273 (48.7%)	101/145 (69.7%)	2.17 (1.09–4.40)	0.03

## Discussion

In our study we aimed to identify the determinants among parents associated with refusal of HPV vaccination for their daughters. We found that, according to reports of parents, limited information provided by the government was the strongest predictor for declining the HPV vaccinations. Although the HPV vaccination campaign included the distribution of invitation letters to families with daughters in the target group and recruitment was supported by a nationwide information campaign targeting health care professionals and the general public [13], these efforts were not sufficient to persuade people to accept the vaccine. Possible explanation, as mentioned before, could include critical media reports that accused the government of emphasizing the advantages rather than the disadvantages of the vaccine. However, it has also been shown that the content of media reports sometimes may lack important information related to the vaccination or the disease [14] which could be misleading.

Another strong determinant for declining the HPV vaccination was a lack of trust that the government would stop vaccination if there were serious side effects. This concern might partly be a consequence of reports about potential associations between certain vaccines and serious adverse events, such as pandemic influenza vaccine and Guillain-Barré syndrome [15]. This suggests that providing information about the management of vaccine side effects may improve the trust in the government as well.

Concerns about the HPV vaccine effectiveness and safety were associated with refusal of the HPV vaccination. One Dutch study showed that those who were not willing to have their children vaccinated said that they would agree to do so after the vaccine had been used for several years [7]. Secondly, it has also been found that 88% of parents said they would be willing to have their children vaccinated if the government approved the vaccine [7]. Although the HPV vaccine was approved by the Dutch government, which means that a list of criteria such as acceptable safety and effectiveness profiles had to be met [16], the actual behavior was not consistent with parents’ reported intentions. Only slightly above 50% of the target population accepted the HPV vaccine. Differences between the intentions and the actual behavior regarding the uptake of the HPV vaccinations may therefore be of interest for future research.

Our findings indicate that parents of vaccinated and unvaccinated girls hold very different views on the severity of HPV infection and the likelihood that their daughters might acquire an HPV infection or cervical cancer and on the question as to whether the information about HPV vaccination was adequate. Parents whose daughters were not vaccinated perceived less risk associated with HPV and cervical cancer. Interestingly they felt, more often, that information about HPV vaccination was not sufficient to make a good decision. Although the entire target group received the same information about the HPV vaccination, these findings suggest that the attitudes towards HPV vaccination were largely influenced by more subjective reasons.

The results of our study show that religious respondents were less likely to accept the vaccine. Another Dutch study also found that voters for religious parties are less likely to accept HPV vaccination [3]. This observation is consistent with the general refusal of childhood vaccination by a group of reformed orthodox living in the Netherlands (http://www.rivm.nl/en/infectious-diseases/topics/nip/). (http://www.zorgatlas.nl/preventie/vaccinaties-en-screening/hpv-cohort-1997-per-gemeente-2010/#breadcrumb). The findings from other countries however provide only limited evidence about the association between religion and HPV vaccine uptake [10,9].

Moreover, preliminary results from other countries show that HPV vaccination coverage largely depends on the type of vaccination program that is implemented. A school-based approach was superior to vaccination programs on-demand through health professionals, the latter being implemented in the Netherlands [13]. The three-dose vaccination coverage in Scotland in the 1996 birth cohort via a school-based HPV vaccination campaign was 86% [17]. It therefore appears that apart from a need for clearer and more transparent messages to the public, different approaches to reach the target population should also be considered.

From the discussions with the stakeholders at the beginning of our study and during the start of the HPV vaccination campaign in the Netherlands it appeared that parents have much influence on the decision as to whether or not to vaccinate their daughters. However, vaccination coverage rates may also be influenced by the girls’ opinions themselves. It has been shown that different information sources may be preferred by girls of different age [18,19]. For example, information provided by health care professionals and mass media (television, the internet) seemed to be a preferred source of information among older teenage girls (15- to 18- or 19-years-old) while younger teenagers (11- or 12- to 14-years-old) had more trust in schools, teachers and family [18,19]. Guidance about HPV and HPV vaccination could therefore be provided through the preferred sources of information for different age groups.

Some limitations of the study need to be addressed. The overall response rate was not high (24%). One of the possible explanations is the two-step response process we employed in which parents were first asked to return a card for participation and only then received a questionnaire. Importantly, the response rate was twice as high among those who received the vaccine versus those who did not (31% versus 16%). This might have introduced bias because those with more positive attitudes towards vaccination were better represented in our study. We expect that the bias introduced more contrast between positive and negative attitudes than in the general population, which agrees with the exceptionally high discriminative value of the predictive model (AUC-value of 0.96). This means that in the general population the role of these determinants is likely to be less important than what we observed in our study. On the other hand, given the fact that some baseline characteristics were comparable to the general Dutch population the sample seemed to mirror the source population of the Netherlands. Our study also assessed a broad spectrum of possible determinants associated with declining HPV vaccination.

The incidence and mortality from cervical cancer in the Netherlands is one of the lowest in Europe. However, since it is the second most common cancer in 18- to 44-years-old women [20], efforts should be made to prevent it. If 70% of the cervical cancers can be prevented by the currently registered HPV vaccines, it could largely reduce physical and psychological disease burden for the females and their families.

## Conclusions

We identified several determinants for the low HPV vaccination uptake. Modifying these determinants might be essential during planning, implementation and continuation of the HPV vaccination programs inside and outside the Netherlands. Furthermore, openness and discussion about the pros and cons of HPV vaccination as well as the use of a variety of communication strategies may be helpful for a more successful implementation of HPV vaccination programs.

## Appendix 1. Knowledge about HPV infection

1. Often HPV does not present with visible symptoms (true).

2. A cervical smear induces cervical HPV infection (false).

3. HPV usually disappears without treatment (true).

4. More sexual partners increase the risk to get HPV infection (true).

5. It is possible to have HPV for a long time without knowing (true).

6. HPV can be transmitted during the sexual contact (true).

7. HPV can cause the cervical cancer (true).

8. Most of the sexually active people at some point get HPV (true).

9. A condom provides 100% protection against HPV (false).

10. If you have HPV, you always know it (false).

## Abbreviations

HPV, Human Papillomavirus; NIP, National Immunization Program; RIVM, Institute for Public Health and the Environment.

## Competing interest

All authors declare to have no competing interest.

## Authors’ contributions

MS conducted the study, collected, analyzed the data and drafted the first version of a manuscript. GG re-analyzed the data and drafted the final version of the manuscript. HWN and ATG contributed to the design of the study and critically reviewed the manuscript. IHD and BW participated in the qualitative assessments and critically reviewed the manuscript. MJP, JCW and JJMvD critically commented on the manuscript. EH contributed to the analysis of the study and critically commented on the manuscript. All authors read and approved the final version of a manuscript.

## Pre-publication history

The pre-publication history for this paper can be accessed here:

http://www.biomedcentral.com/1471-2458/12/498/prepub

## Supplementary Material

Additional file 1 Questionnaire. (DOC 186 kb) Click here for file
