# Supplementary material for: Comparatively low attendance during Human Papillomavirus catch-up vaccination among teenage girls in the Netherlands: Insights from a behavioral survey among parents
Source: BMC Public Health. 2012 Jul 2;12:498. doi: 10.1186/1471-2458-12-498 (PMC3461412; doi:10.1186/1471-2458-12-498)
Supplement: Additional file 1 — Questionnaire. (DOC 186 kb) [file 1471-2458-12-498-S1.doc]

| **General instruction**  *Please tick off the boxes as clearly as possible with a blue or black pen. For every question you can fill out one option, unless stated otherwise.* |
| --- |

| A. General questions | | | |
| --- | --- | --- | --- |
|  | What is your gender? | - Man - Woman | |
|  | What is your age? | Years | |
|  | What is your marital status? | - Single - Married - Living together - Divorced/ separated - Widowed | |
|  | What is the highest education that you completed with a certificate? | - No education completed - Primary education - Lower vocational education / Initial professional education - Lower general secondary education - Intermediate vocational education - Higher general secondary education / Pre-university education - Higher vocational education / Higher education (university) | |
|  | What is your religion? | - None - Christian Catholic - Protestant - Muslim - Other, namely . | |
|  | What is your country of birth? | - The Netherlands - Other European country - Other, namely . | |
|  | | | |
|  | Do you know someone with an abnormal cervical smear or cervical cancer in your family or acquaintances? | | - Yes - No - Don’t know - Do not wish to answer |
|  | Do you/ your partner participate in cervical screening (making of cervical smears)? | | - Yes - No - Not applicable - Do not wish to answer |

| B. Vaccinations in general (While responding the questions we want to ask you to think about ***your daughter*** who received a ***call for the HPV vaccination***) | | |
| --- | --- | --- |
|  | Did your daughter receive the regular/ recommended vaccination of the National Institute Program? | - Yes, all of them - Yes, not all of them - No - Don’t know |
|  | Did your daughter receive the MMR vaccination at the age of 9 years? | - Yes - No   x   - Don’t know |
|  | Have any of your children ever had a **bad reaction/ side effect** after vaccination with one of the vaccines of the National Institute Program? | |
|  | - No - Yes, mild side effect (for example local reaction, painful arm, fever) - Yes, severe side effect (for example seizure, severe anaphylactic reaction) | |
|  | Have you ever **regretted** a decision to get one of your children vaccinated? | |
|  | - No - Yes, reason: . | |

|  | Please read each of the statements below and show how much you agree or disagree with the statement by ticking the appropriate box. | | | | | | |
| --- | --- | --- | --- | --- | --- | --- | --- |
|  |  | | Strongly disagree | Disagree | Neutral | Agree | Strongly agree |
|  |  | It’s likely that my daughter gets infected with HPV some day |  |  |  |  |  |
|  |  | I believe HPV can be extremely harmful |  |  |  |  |  |
|  |  | It’s possible that my daughter gets infected with HPV some day |  |  |  |  |  |
|  |  | I believe that HPV can cause cervical cancer |  |  |  |  |  |
|  |  | It’s possible that my daughter gets cervical cancer in the future |  |  |  |  |  |
|  |  | I believe that cervical cancer is a serious disease |  |  |  |  |  |
|  |  | It’s likely that my daughter gets cervical cancer in the future |  |  |  |  |  |

| C. Questions about HPV and HPV vaccination (While responding the questions we want to ask you to think about ***your daughter*** who received a ***call for the HPV vaccination***) | | | | | |
| --- | --- | --- | --- | --- | --- |
|  | Please read each of the statements below and show if you think the statement is true or false | | | | |
|  |  | | True | False | Don’t know |
|  |  | Often HPV does not present with visible symptoms |  |  |  |
|  |  | A cervical smear induces cervical HPV infection |  |  |  |
|  |  | HPV usually disappears without treatment |  |  |  |
|  |  | More sexual partners increase the risk to get HPV infection |  |  |  |
|  |  | It is possible to have HPV for a long time without knowing |  |  |  |
|  |  | HPV can be transmitted during the sexual contact |  |  |  |
|  |  | HPV can cause the cervical cancer |  |  |  |
|  |  | Most of the sexually active people at some point get HPV |  |  |  |
|  |  | A condom provides 100% protection against HPV |  |  |  |
|  |  | If you have HPV, you always know it |  |  |  |

|  | Please read each of the statements below and show how much you agree or disagree with the statements about HPV vaccination by ticking the appropriate box. | | | | | |
| --- | --- | --- | --- | --- | --- | --- |
|  |  | | Strongly disagree | Disagree | Agree | Strongly agree |
|  |  | I'm very positive about the HPV vaccine |  |  |  |  |
|  |  | I want to be sure and didn’t get my daughter vaccinated |  |  |  |  |
|  |  | Cervical cancer is not something I'm worried about right now for my daughter |  |  |  |  |
|  |  | I feel I had enough information to make a good decision |  |  |  |  |
|  |  | I would strongly disapprove if my daughter would be sexually active at this age |  |  |  |  |
|  |  | | Strongly disagree | Disagree | Agree | Strongly agree |
|  |  | I'm very worried about the side effects of the HPV vaccination |  |  |  |  |
|  |  | Vaccines are very effective in preventing diseases |  |  |  |  |
|  |  | HPV is not that serious to get vaccinated for |  |  |  |  |
|  |  | Doctors do not take parents serious about what they state about side effects of vaccinations |  |  |  |  |
|  |  | There are already too many vaccines in the Dutch vaccination program |  |  |  |  |
|  |  | I don’t believe/ trust that the government would stop vaccinations if there was evidence of serious side effects |  |  |  |  |
|  |  | I think the information about the vaccine provided by the government was very clear |  |  |  |  |
|  |  | We know way too little about the effects of the vaccine |  |  |  |  |
|  |  | It's very important that my children receive all their vaccinations |  |  |  |  |
|  |  | We already know a lot about the side effects of the vaccine |  |  |  |  |
|  |  | I think the HPV vaccination is unnecessary because there already is a screening program |  |  |  |  |
|  |  | Other girls might need the vaccination, but my daughter won't |  |  |  |  |
|  |  | I will do everything to prevent my daughter getting cervical cancer |  |  |  |  |
|  |  | I would had have more information to make a good decision |  |  |  |  |
|  |  | Having the HPV vaccination might make girls more likely to have sex |  |  |  |  |
|  |  | The government is strongly influenced by the vaccine producers |  |  |  |  |
|  |  | | Strongly disagree | Disagree | Agree | Strongly agree |
|  |  | There were enough locations to get the vaccination |  |  |  |  |
|  |  | Girls who had the HPV vaccination would be more likely to have unprotected sex |  |  |  |  |
|  |  | I think it's good that the HPV vaccine exists, but not at this age |  |  |  |  |
|  |  | It was very clear when my daughter could get the HPV vaccine |  |  |  |  |
|  |  | I think the information about the vaccine provided by the government was very limited/ biased |  |  |  |  |
|  |  | I think my daughter is very capable to make her own decision about taking the vaccination |  |  |  |  |
|  |  | I would get my daughter vaccinated if the vaccine wasn't only for girls but also for boys |  |  |  |  |

| D. Information services  Caution! Question 16 & 17 are the same, question 16 is about ***your*** decision, and question 17 is about ***your daughter’s*** decision! | | | |
| --- | --- | --- | --- |
|  | Which of the sources written below was most important in ***your*** decision?  Please arrange the sources written below in the way of importance (number 1 till 12 where 1 was most important and 12 least important for the decision) | | |
|  |  | | Arrangement 1- 12 |
|  |  | Your daughter |  |
|  |  | TV (news, opinion broadcasting) |  |
|  |  | Internet (website information) |  |
|  |  | Facebook, myspace (peer groups) |  |
|  |  | Family |  |
|  |  | Friends/ acquaintances |  |
|  |  | (House) doctor |  |
|  |  | School |  |
|  |  | Brochure GGD/ RIVM |  |
|  |  | Information meeting |  |
|  |  | Newspaper |  |
|  |  | Radio |  |

| CAUTION, this question is about ***your daughter’s*** decision | | | |
| --- | --- | --- | --- |
|  | Which of the sources written below was most important in ***your daughter’s*** decision?  Please arrange the sources written below in the way of importance (number 1 till 12 where 1 was most important and 12 least important for the decision) (you may do this in agreement with your daughter) | | |
|  |  | | Arrangement 1- 12 |
|  |  | You/ your partner |  |
|  |  | TV (news, opinion broadcasting) |  |
|  |  | Internet (website information) |  |
|  |  | Facebook, MySpace (peer groups) |  |
|  |  | Family |  |
|  |  | Friends/ acquaintances |  |
|  |  | (House) doctor |  |
|  |  | School |  |
|  |  | Brochure GGD/ RIVM |  |
|  |  | Information meeting |  |
|  |  | Newspaper |  |
|  |  | Radio |  |

| Caution! Question 18 & 19 are the same, question 18 is about websites ***you*** used for gathering information, and question 19 is about websites your daughter used for gathering information! | | | | |
| --- | --- | --- | --- | --- |
|  | Name several websites ***you*** used for gathering information (max. 3) | | | |
|  | I didn’t use any website | |  | |
|  | Yes, I used following websites: | |  | |
|  |  | .  . | | |
|  |  | .  . | | |
|  |  | .  . | | |
|  | Name several websites ***your daughter*** used for gathering information (max. 3) | | | |
|  | My daughter didn’t use any website | | |  |
|  | Yes, following websites: | | |  |
|  |  | .  . | | |
|  |  | .  . | | |
|  |  | .  . | | |

| E. Ethical and economic considerations | | | | | | | |
| --- | --- | --- | --- | --- | --- | --- | --- |
|  | As of which age you feel comfortable discussing the following subjects with your daughter? | | | | | | |
|  |  | | Before the age of 9years | As of 9 years | As of 12 years | As of 15 years | Never |
|  |  | Cancer in general |  |  |  |  |  |
|  |  | Cervical cancer |  |  |  |  |  |
|  |  | Sex in general |  |  |  |  |  |
|  |  | Sexual transmitted diseases |  |  |  |  |  |
|  |  | Purpose of vaccinations in general |  |  |  |  |  |
|  |  | Human papillomavirus (HPV) |  |  |  |  |  |
|  |  | HPV-vaccination |  |  |  |  |  |
|  | Please indicate at which age you think girls should be vaccinated against HPV | | years | | | | |
|  | How much would you be prepared to pay for the set of 3 vaccinations (total price) if these were not for free? | | euro | | | | |
|  | Did you consider vaccinating your older daughter(s) on own expenses (outside the National Immunization Programme)? | | - Yes, I already did this - Yes, I did not do it yet but I’m planning to do this - Yes, if it was for free - No, even if it was for free - Not applicable (I don’t have older daughter(s)) | | | | |

| F. Finishing questions | | |
| --- | --- | --- |
|  | Did your daughter receive the HPV vaccination? | - Yes - No - Not yet, we are considering to do it - I don’t know |
|  | Who took the final decision to take or not take the vaccine? | - Your daughter - You/ your partner - Together |
|  | Did you agree with the final decision of your daughter? | - Yes - No - I don’t know |
|  | Did your daughter already receive her second injection? | - Yes - No - I don’t know |
|  | Would you vaccinate your daughter for other diseases that are sexually transmitted in the future if they would come available (for example Hepatitis B, HIV/AIDS)? | - Certainly yes - Certainly not - I’m not sure yet |

| Space for comments |
| --- |
|  |
